# Age-dependent effects of Delaire facemask therapy for class III malocclusion: Impact on maxillary sutures and palatal morphology

**DOI:** 10.1007/s00056-024-00564-9

**Published:** 2024-12-23

**Authors:** Gero Stefan Michael Kinzinger, Jan Hourfar, Joana Nanina Sommer, Jörg Alexander Lisson

**Affiliations:** Department of Orthodontics, Saarland University, 66424 Homburg/Saar, Germany

**Keywords:** Maxilla, Transverse palatine suture, Pterygopalatomaxillary sutures, Cone-beam computed tomography, Cast analysis, Oberkiefer, Sutura palatina transversa, Pterygopalatomaxilläre Suturen, Digitale Volumentomographie (DVT), Modellanalyse

## Abstract

**Background and aim:**

Treatment effects of a combined rapid maxillary expansion (RME) and Delaire facemask (DFM) therapy have so far only been scientifically investigated through cephalometric analyses. The combination of cephalometric, dental cast and cone-beam computed tomography (CBCT) scan analysis was not yet used for investigating morphologic changes of the tooth-bearing palate. The aim of the present study was to determine whether patient age at treatment begin has an influence upon palatal length changes after RME/DFM therapy, and to what extent transverse palatal sutures contribute to these.

**Patients and methods:**

In *n* = 6 patients (min 10.5 years, max 14.7 years) from a total group of *n* = 40, CBCT datasets showing all palatal sutures were visually assessed, and palatal morphology was compared with a dental cast analysis. In addition, lateral cephalograms and dental casts of *n* = 40 patients were divided into two groups (PG1: < 12 years, *n* = 20; PG2: ≥ 12 years, *n* = 20), and an analysis was performed to investigate changes in the tooth-bearing palate after RME/DFM treatment.

**Results:**

The CBCT analysis showed that the median and transverse palatine sutures were always open. On the contrary, the pterygopalatomaxillary sutures were partially open only in the youngest patients. The transverse palatal suture showed age-dependent morphologic changes in the transverse and sagittal planes. The changes of the palatal width and length show clear differences between the two younger and the four older patients in the corresponding dental cast analysis. The cephalometric analysis showed that a significant improvement of the sagittal jaw relation due to ventral displacement of the maxilla during treatment occurred only in younger patients (< 12 years) despite similar initial findings in both patient groups. The dental cast analysis also revealed that changes are age-dependent: In PG1, the width increases posteriorly more than anteriorly; in PG2, this is reversed. The length increases are always significant in both patient groups, whereby the anterior, posterior, and total amounts are greater in PG1 than in PG2. In relative terms, the increases in both groups are greater posteriorly than anteriorly. There is a significant difference between the groups posteriorly and overall.

**Conclusions:**

Morphological changes of the sutures appear to have a decisive influence on the success of RME/DFM therapy. The age-dependent reactions of pterygopalatomaxillary and transverse palatine sutures represent a further main therapeutic effect of DFM treatment in addition to those described by Delaire and explain the different changes in palate length before and after the age of 12. If the maximum effect of RME/DFM treatment is desired, it should be started before the age of 12. Treatment success is age-dependent.

## Abstract (de)

**Hintergrund und Ziel:**

Die Behandlungseffekte einer kombinierten Therapie aus „rapid maxillary expansion“ (RME) und „facemask“ nach Delaire (DFM) wurden bislang nur durch kephalometrische Studien wissenschaftlich überprüft. Durch diese Therapie veranlasste morphologische Veränderungen des Gaumens sind dagegen bislang nicht kombiniert durch kephalometrische, Modell- und DVT(digitale Volumentomographie)-Analysen untersucht worden. Ziel der vorliegenden Studie war es festzustellen, ob das Alter der Patienten bei Behandlungsbeginn einen Einfluss auf die Längenveränderungen des Gaumens nach RME/DFM-Therapie hat und inwieweit die quer verlaufenden palatinalen Suturen dazu beitragen.

**Patienten und Methoden:**

Bei *n* = 6 Patienten (min. 10,5 Jahre, max. 14,7 Jahre) aus einer Gesamtgruppe von *n* = 40 wurden DVT-Datensätze im Bereich aller palatinalen Suturen visuell befundet und die Gaumenmorphologie mit einer Modell-Analyse abgeglichen. Zusätzlich wurden Fernröntgenseitenbilder und Modelle von *n* = 40 Patienten in zwei Gruppen unterteilt (PG1: < 12. Lebensjahr, *n* = 20; PG2: ≥ 12, *n* = 20), und es wurde eine Analyse durchgeführt, um Veränderungen des zahntragenden Gaumens nach der RME/DFM-Behandlung zu beurteilen.

**Ergebnisse:**

Die DVT-Analyse zeigte, dass die Sutura palatina mediana und die Sutura palatina transversa immer geöffnet waren. Die pterygopalatomaxillären Suturen waren dagegen nur bei den jüngsten Patienten partiell geöffnet. Die Sutura palatina transversa zeigt in der Transversal- sowie in der Sagittalebene altersabhängige morphologische Veränderungen. Die Werte der Gaumenbreiten- und Gaumenlängenveränderungen zeigen bei der korrespondierenden Modellanalyse deutliche Unterschiede zwischen den beiden jüngeren und den 4 älteren Patienten. Die FRS-Analyse zeigt vergleichbare Anomalien in beiden Patientengruppen zu Behandlungsbeginn, eine signifikante Verbesserung der mesiobasalen Kieferbasenrelation durch sagittale Ventralverlagerung der Maxilla unter der Behandlung aber nur bei den jüngeren Patienten (< 12. Lebensjahr). In der Modellstudie sind die Veränderungen ebenfalls altersabhängig: Bei PG1 nimmt die Breite im posterioren Bereich mehr als anterior zu, bei PG2 ist dies umgekehrt. Die Längenzunahmen sind in beiden Patientengruppen immer signifikant, wobei sowohl anterior, posterior als auch gesamt die Beträge bei PG1 größer sind als bei PG2. Relativ betrachtet sind die Zunahmen in beiden Gruppen posterior größer als anterior. Ein signifikanter Unterschied zwischen den Gruppenwerten besteht posterior und gesamt.

**Schlussfolgerungen:**

Morphologische Veränderungen der Suturen scheinen einen entscheidenden Einfluss auf den Erfolg der RME/DFM-Therapie zu haben. Die altersabhängigen Reaktionen der pterygopalatomaxillären Suturen und der Sutura palatina transversa stellen einen weiteren, zusätzlich zu den von Delaire beschriebenen therapeutischen Haupteffekt der DFM-Behandlung dar und erklären die unterschiedlichen Gaumenlängenveränderungen vor und nach dem 12. Lebensjahr. Will man den maximalen Effekt der RME/DFM-Behandlung erzielen, sollte sie vor dem 12. Lebensjahr begonnen werden. Der Behandlungserfolg ist altersabhängig.

## Introduction

Skeletal Angle class III malocclusions are among the most challenging orthodontic problems [1]. Although class III malocclusion treatment often focuses solely on the mandible as the primary etiologic cause [2–4], study results suggest that most class III malocclusions involve maxillary micrognathia and/or retrognathia in combination with a normal or only slightly prognathic mandible [5–10].

If transverse and sagittal deficits of the maxilla are present, treatment with a combination of rapid maxillary expansion (RME) and Delaire facemask (DFM) is useful [11–13]. Rapid maxillary expansion was first described by Angell [14] in 1860, and therapy with a mask to protract the maxilla was first described by Potpeschnigg [15] in 1875. Both therapies are, thus, among the oldest treatment modalities in orthodontics.

The therapeutic effects of RME have been extensively investigated. These studies mainly focused on skeletal and dental effects including side effects [16–21], and the effects on the median palatal suture [22–28].

The treatment effects of the facemask were evaluated in the 1970s by Delaire et al. [29–31] who described two different effects on the maxilla: forward pivoting in the frontomaxillary joint and forward movement of the maxillary alveolar and dental arches. However, Delaire et al. [30, 31] stated that forward pivoting of the maxilla is not possible after 12 years of age. Baccetti et al. [32] and Franchi et al. [33] additionally described the effect of disarticulation of the palatal bone from the pterygoid process, but this occurs only during early mixed dentition. Thus, the effects of a facemask therapy are probably age dependent.

Especially morphological changes of the tooth-bearing palate are significant for orthodontic treatment outcome. Recent studies evaluated the effects of a dentally anchored RME on the morphology of the tooth-bearing palate for the first time. Kinzinger et al. [34] demonstrated that the therapeutic effects of RME on palatal morphology varied depending on dentition stages. In another study, the authors subdivided the patients according to chronological age [35]. They concluded that maxillary expansion after RME occurs parallel in children up to 10 years of age, whereas it tends to be V‑shaped and less pronounced with increasing age and especially in adolescents older than 12 years. Therefore, they concluded that the treatment success of RME depends significantly on patient age at treatment begin. In addition to an age-progressive rigidity of the pterygopalatomaxillary suture, morphological changes of the transverse palatine suture during growth seem to be the main reason for this.

It remains unclear whether different age-dependent morphological changes in the sutures have a decisive influence on the forward movement of the maxilla through DFM treatment besides the effects of an RME.

## Aims

The aims of this study were to answer the following questions:

- Are changes in palatal width and length caused by the combination of RME and DFM age dependent?
- Do cone-beam computed tomography (CBCT) datasets provide insights about causes of age-progressive changes?
- Do morphological changes in the transverse palatal sutures play a role in the treatment effects seen on dental casts?
- Are there age-related sutural effects besides the facemask effects described by Delaire?
- Can the effects of RME be differentiated from those of DFM in combination treatment?

## Materials and methods

The study setup first used a limited number of CBCT datasets for an initial comparison with the results of a corresponding dental cast analysis. Second, a comprehensive cephalometric and dental cast analysis was carried out to quantify the therapeutic effects of a combined RME/DFM treatment on the morphology of the tooth-bearing palate. The combination of the results was then used to examine possible influences of the transverse palatal and pterygopalatomaxillary sutures on the morphological changes.

### Patients

Of 54 patients treated with a combined RME/DFM appliance for maxillary expansion and advancement, 40 patients (21 male, 19 female) were included in the study.

The inclusion criteria were the following:

- Skeletal Angle class III malocclusion with maxillary retrognathia and mesiobasal jaw relation,
- No previous orthodontic treatment,
- Caucasian origin (visual inspection),
- Anterior and posterior maxillary arch constriction ≥ 3 mm, and
- Dental casts and lateral cephalograms before (T1) and immediately after (T2) RME/DFM.

In all, 6 patients from the total group received CBCT scans shortly before removal of the RME/DFM appliance (DVT Carestream CS 8100 3D appliance, Carestream Dental LLC, Atlanta, GA, USA). The indication for the scans was to clarify possible ectopia or hyperodontia. CBCT scans prior to treatment were unavailable.

Based on the age at T1, 40 patients were divided into two equal groups: Patients up to 12 years were assigned to group 1 (PG1) and from 12 years to group 2 (PG2). The youngest patient was 7.0 years old at T1, the oldest 15.5 years. The mean age was 11.61 ± 2.05 years (PG1: 9.93 ± 1.36 years, PG2: 13.29 ± 1.03 years, *p* < 0.001). The allocation of the total group to the equally sized patient groups was carried out in such a way that the number of Hyrax screw activations (mean 22.83 ± 3.75; PG1: 22.80 ± 3.91; PG2: 22.85 ± 3.54, *p* = 0.967) and the insertion duration of the RME/DFM (mean 6.57 ± 1.19 months, PG1: 6.56 ± 1.36 months, PG2: 6.85 ± 1.13 months, *p* = 0.971) matched as closely as possible.

### RME/DFM appliance

All patients received an RME appliance with dental anchorage. This appliance included a Hyrax screw (palatal screw type S, Forestadent, Pforzheim, Germany with a lift height of 0.2 mm), and was attached with two occlusal rests on the first premolars or deciduous molars, respectively, and banded to the first molars. The molar bands included vestibular hooks. Those were used to take up the elastics (2 × 14 oz or 2 × 400 g, blue whale, article no. 000-143, American Orthodontics, Sheboygan, WI, USA) between the RME and the DFM (article no. GM 1001/1002, Orthana GmbH, Recklinghausen, Germany). The RME screw was activated twice daily until the desired dental arch expansion including moderate overcorrection was achieved. Patients were instructed to start wearing the DFM after the last screw activation, and to wear it with the elastics angulated 30° caudally for 10–12 h/day.

### CBCT scan analysis

The CBCT datasets of 6 patients were first used to verify whether the incisive suture, the median palatine suture, the transverse palatine suture, and the pterygopalatomaxillary sutures are detectable and, if present, closed, partially open or fully open. The shape and course of the transverse palatine suture were determined in the transverse and sagittal planes, and of the pterygopalatomaxillary sutures in the transverse plane (Table 1). Finally, an exemplary 3D reconstruction was created from CBCT datasets in 2 patients.

**Table 1:** Patients with a cone-beam computed tomography (CBCT) scan Patienten mit digitaler Volumentomographie (DVT)

Patient no.	1	2	3	4	5	6
*Age* *(years)*	10.5	10.10	12.5	12.8	13.7	14.7
*Gender (m/f)*	m	f	m	f	f	f
*Wearing time FM/RME (months)*	6.5	6	6	7.5	7	6
*Hyrax screw activation*	19	19	19	23	15	20
*Power FM (oz/grams)*	2 × 14/2 × 400	2 × 14/2 × 400	2 × 14/2 × 400	2 × 14/2 × 400	2 × 14/2 × 400	2 × 14/2 × 400
*Visual suture inspection (open/partially open/closed/undetectable)*						
Sutura incisiva	Undetec.	Undetec.	Undetec.	Undetec.	Undetec.	Undetec.
Median palatal suture	open	open	open	open	open	open
Transverse palatal suture	open	open	open	open	open	open
Pterygopalatomaxillary suture right/left	part.o./part.o	part.o./part.o	clsd./clsd.	clsd./clsd.	clsd./clsd.	clsd./clsd.
*Visual suture inspection* Transverse palatal suture shape/course in the transverse plane	Anterior bulging at midline, symmetrical	Anterior bulging at midline, symmetrical	Posterior bulging at midline, symmetrical	Posterior bulging at midline, symmetrical	Straight, symmetrical	Straight, asymmetrical
*Visual suture inspection* Transverse palatal suture shape/course in the sagittal plane	Vertical line from cranial to caudal	Vertical line from cranial to caudal	Oblique line from ventral-cranial to dorsal-caudal	Oblique line from ventral-cranial to dorsal-caudal	Oblique line from ventral-cranial to dorsal-caudal	Oblique line from ventral-cranial to dorsal-caudal
*Visual suture inspection* Pterygopalatine suture oriented relatively perpendicularly or angled to FM movement in the transverse plane	Angled	Angled	Angled	Perpendicularly	Angled	Perpendicularly
*Cast measurements (mm) transverse plane*						
Width anterior (4-4)						
Dental	4.3	4.4	3.5	3.7	3.9	3.8
Gingival–alveolar	3.6	3.6	3.4	3.6	3.4	3.1
Skeletal–basal	1.7	2.0	1.8	1.6	1.2	1.8
Width posterior (6-6)						
Dental	4.8	4.2	3.5	3.5	3.2	2.7
Gingival–alveolar	3.8	3.5	3.3	3.5	2.7	2.2
Skeletal–basal	1.9	1.8	1.6	1.3	0.9	1.2
Ratio a/p						
Dental	0.89	1.05	1.00	1.06	1.22	1.40
Gingival–alveolar	0.95	1.03	1.03	1.03	1.26	1.41
Skeletal–basal	0.89	1.11	1.12	1.23	1.33	1.50
*Cast measurements (mm) sagittal plane*						
Palate length anterior						
median	1.2	1.2	0.4	0.6	0.3	0.2
5 mm right	1.2	1.1	0.4	0.7	0.3	0.2
5 mm left	1.1	1.2	0.4	0.6	0.3	0.2
Palate length posterior						
median	2.4	2.3	1.9	1.3	1.3	0.6
5 mm right	2.3	2.3	2.0	1.2	1.4	0.7
5 mm left	2.5	2.2	2.0	1.3	1.5	0.7
Palate length total						
median	3.6	3.5	2.3	1.9	1.6	0.8
5 mm right	3.5	3.4	2.4	1.9	1.7	0.9
5 mm left	3.5	3.4	2.4	1.9	1.7	0.9

age, gender, mean FM/RME wear time, number of Hyrax screw activations, visual inspection CBCT at T2, cast measurement ∆T2–T1 (mm)

*undetec.* undetectable, *part.o.* partially open, *clsd.* Closed, *a/p* anterior/posterior, *m* male, *f* female, *RME* rapid maxillary expansion, *FM* Delaire facemask (mm)

### Cephalometric analysis

**Fig. 1:**
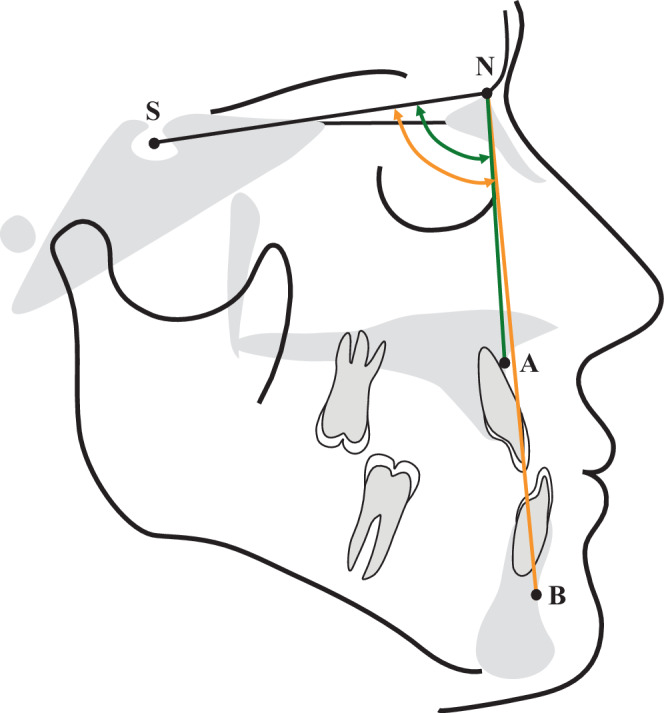
Cephalometric analysis: sagittal skeletal angular measurements Kephalometrische Analyse: sagittal-skelettale Winkelmessungen

Selected sagittal parameters (SNA, SNB, and ANB, Fig. 1) were measured on lateral cephalograms of 40 patients at T1 and T2 to determine the type of class III anomaly existing at the start of treatment and to ensure comparability of the patient groups, and to be able to evaluate differential therapeutic effects at the end of treatment.

### Dental cast analysis

In all, 80 maxillary dental casts were digitized and traced before insertion (T1) and immediately after removal of the appliance (T2).

**Fig. 2:**
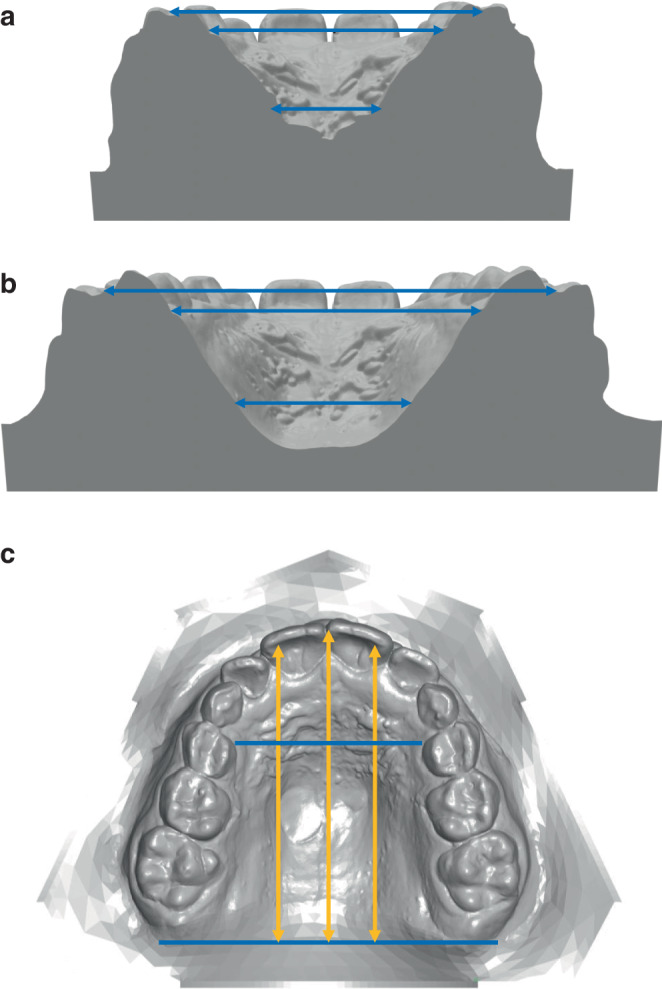
Cast analysis: quantification of palatal widths at the level of the Pont measuring points anterior (**a**) and posterior (**b**). Analysis of palatal lengths (**c**) in the sagittal plane Modellanalyse: Quantifizierung der Gaumenbreiten in der Transversalebene auf Höhe der Pont-Messpunkte anterior (**a**) und posterior (**b**). Analyse der Gaumenlängen (**c**) in der Sagittalebene

The dental arch width was measured according to Pont [36] anteriorly at the first premolars or deciduous molars and posteriorly at the first permanent molars. The palatal width was measured between the most coronal points of the gingival margin at the first premolars or deciduous molars and the first permanent molars (gingival–alveolar plane). Starting from this plane and ascending cranially, the width was also determined 6 mm above (skeletal–basal plane; Fig. 2a, b). On these three vertical planes (dental, gingival–alveolar, and skeletal–basal), the ratio of the width was determined from both individual and mean values anterior/posterior to analyze the type of transverse expansion (values < 1 = inverse V‑shaped/delta-shaped; 0 = parallel, > 1 = V-shaped/triangular). The palatal depth/length in the sagittal plane was measured from the exit of the 3rd pair of palatal folds on the raphe median plane median and 5 mm right and left paramedian and anterior to the incisors and posterior to the tuber plane, respectively (Fig. 2c). The respective total palatal lengths were calculated as the sum of the two partial distances.

### Comparison of CBCT data and cast analyses

**Fig. 3:**
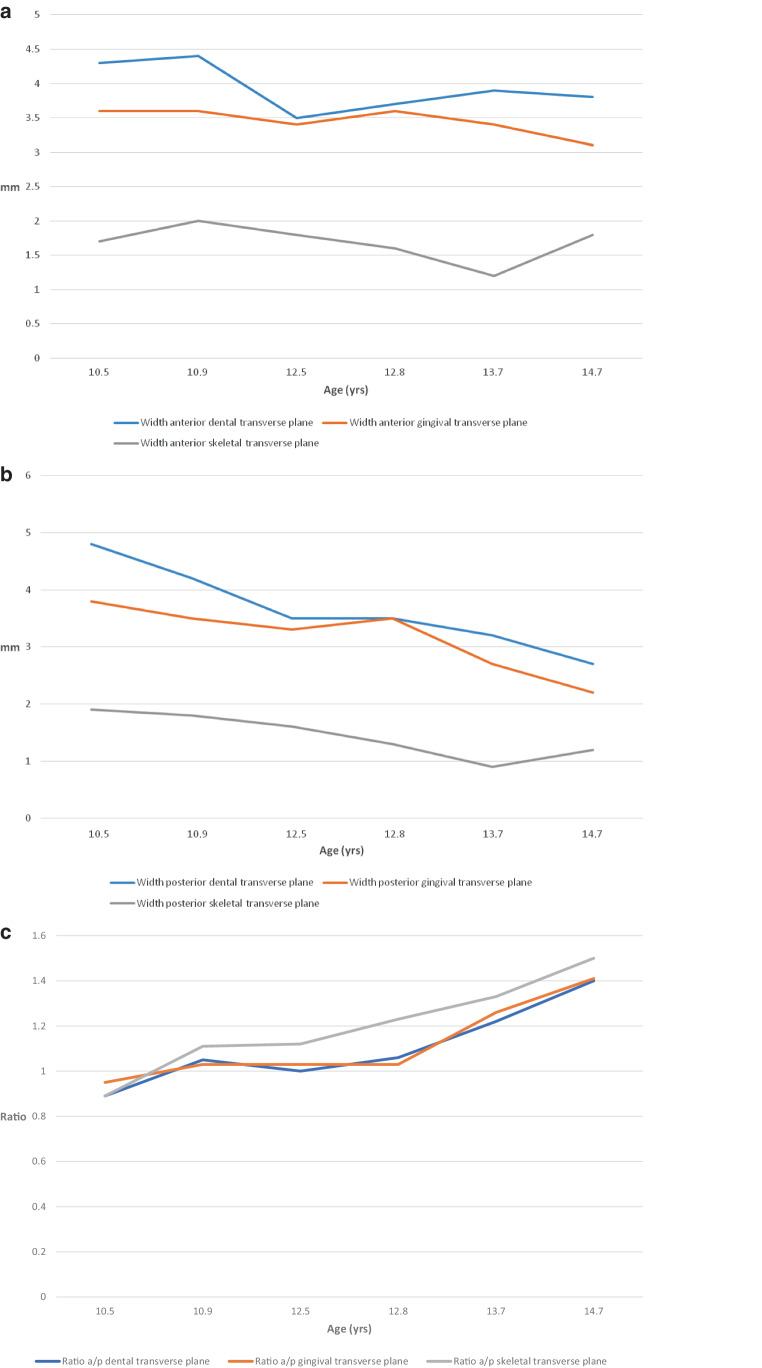
Graphical representation of palatal widths anterior (**a**), posterior (**b**), and anteroposterior (a/p) ratio (**c**). The individual values were calculated on the defined levels on the dental casts and plotted as a function of patient age between 10.5 and 14.7 years Graphische Darstellung der Gaumenbreiten anterior (**a**), posterior (**b**) und der Ratio-Werte a/p (**c**). Die einzelnen Werte wurden auf den definierten Ebenen an den Modellen berechnet und als Funktion des Patientenalters zwischen 10,5 und 14,7 Jahren aufgetragen

**Fig. 4:**
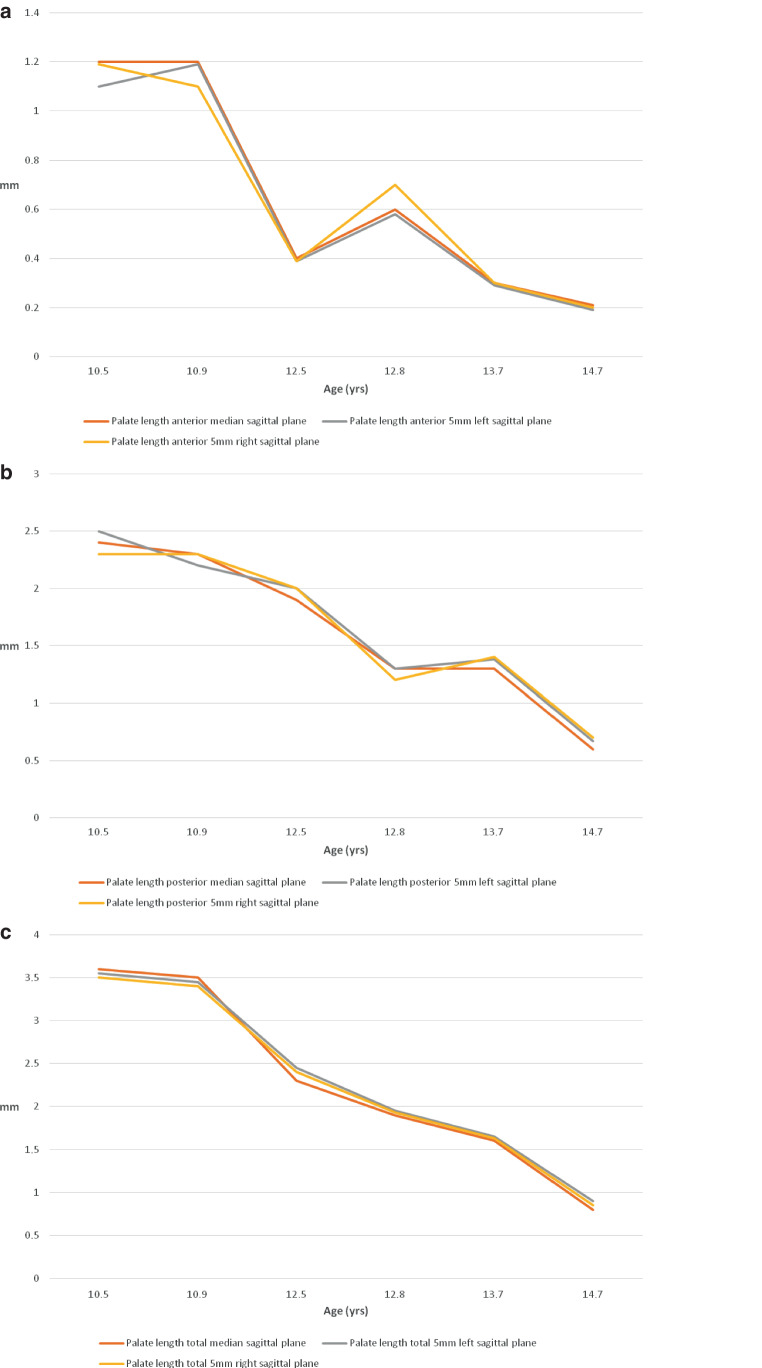
Graphical representation of palatal lengths anterior (**a**), posterior (**b**) and overall (**c**). The individual values were calculated on the defined levels on the dental casts and plotted as a function of patient age between 10.5 and 14.7 years Graphische Darstellung der Gaumenlängen anterior (**a**), posterior (**b**) und gesamt (**c**). Die einzelnen Werte wurden auf den definierten Ebenen an den Modellen berechnet und als Funktion des Patientenalters zwischen 10,5 und 14,7 Jahren aufgetragen

To enable a comparison of the visual findings from the CBCT data with the results of the width and length measurements on dental casts (Table 1), the data obtained from the cast analysis were graphically displayed for *n* = 6 patients (Figs. 3 and 4).

### Statistics and error of the method

Normal distribution was evaluated with the Shapiro–Wilk test. Treatment-associated changes in variables were analyzed for intragroup comparisons using the paired t‑test. Differences between the groups were assessed with the analysis of variance (ANOVA). Post hoc testing was performed using the Tukey test. Homogeneity of variance was confirmed through the Levene test. Mean and standard deviation as well as confidence interval were reported for each variable. Statistical significance was assumed for *p*-values < 0.05.

To determine the combined method error (ME) according to Dahlberg [37], 25% of the casts and lateral cephalograms were randomly selected after a period of 3 months and measured again by the same investigator. The method error for all measurements was calculated with the formula ME = √(∑d^2^/2n) to determine the validity of the measurement method, where d is the difference between two measurement results and *n* is the number of duplicate measurements. The ME in the present study was < 1 for all measurements.

## Results

### Visual assessment of CBCT datasets and corresponding cast analysis (Table 1)

The inspection of the sutures in the CBCT scans revealed that the median and the transverse palatine sutures were open in all 6 patients. The pterygopalatomaxillary sutures were only partially open at the lateral pterygoid processes in the two youngest patients (10.5 and 10.10 years), otherwise they were closed. The incisive suture was without exception not detectable.

In the transverse plane, the transverse palatine suture is curved anteriorly in the two youngest patients and posteriorly in the two 12-year-old patients. In the oldest patients, it describes a straight course with a symmetrical exit in the 13.7-year-old patient and an asymmetrical exit from the median palatine suture in the 14.7-year-old patient. In the sagittal plane, the course is straight on both sides in the two youngest patients, vertically from cranial to caudal. In contrast, the transverse palatine suture of the four older patients describes an oblique course from ventral–cranial to dorsal–caudal. Colak et al. [38] described the possible positions of the pterygopalatomaxillary sutures related to maxillary movement after RME. Based on their description, the sutures are oriented perpendicularly in 2 patients and angulated in 4 patients relative to the facemask traction vector.

In the transverse plane, the cast measurement shows that an almost parallel expansion anteriorly and posteriorly at all levels occurs in the youngest patients. With increasing age, there is less expansion from anterior to posterior and, thus, a V-shaped expansion of the palate. The age-dependent different expansion modes were confirmed by the anterior/posterior ratio: values around 1 proved a parallel expansion of the palate for the younger patients and a V-shaped one with increasing age (Fig. 3c).

In the sagittal plane, the anterior, posterior and overall length increases are greatest in the two 10-year-olds. In the four patients aged 12 and above, significantly lower values were recorded for all measurements. In particular, the graphical representation of the total length visualizes the different increases in length depending on age (Fig. 4c).

### Comparison of the CBCT analyses of three patients of different ages (Figs. 5, 6, and 7)

The CBCT showed open median palatal and transverse sutures in all patients. Some of the median palatal sutures are showing signs of regeneration because of the delayed recording of the datasets (4–6 months) after completion of rapid maxillary expansion.

**Fig. 5:**
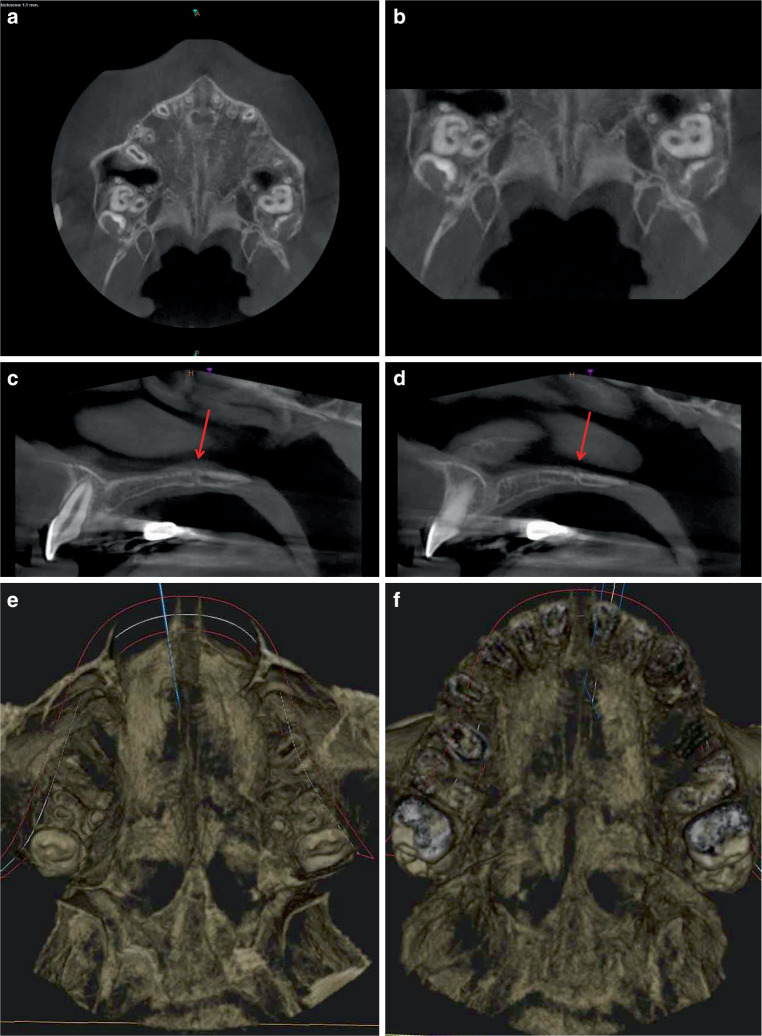
Horizontal (**a**, **b**) and sagittal slices (**c**, **d**) as well as 3D reconstructions with views from cranial and caudal (**e**, **f**) after combined rapid maxillary expansion/Delaire facemask (RME/DFM) treatment from a cone-beam computed tomography (CBCT) scan of a 10.10-year-old girl. **a**, **b** Slice (overview and detail) with open median and open anteriorly curved transverse palatal suture and partially open pterygopalatomaxillary sutures. **c**, **d** Both right and left lateral sagittal planes show a widely open transverse palatal suture (red arrows) with a straight, vertical course from cranial to caudal. **e**, **f** Parallel opening of the median palatal suture and wide open transverse palatal suture from cranial (**e**) and caudal (**f**). Clear quadripartition of the bony palate Horizontale (**a**, **b**) und sagittale Schichten (**c**, **d**) sowie 3‑D-Rekonstruktionen mit Ansichten von kranial und kaudal (**e**, **f**) nach kombinierter RME/DFM(„rapid maxillary expansion/Delaire facemask“)-Behandlung aus der DVT (digitale Volumentomographie) eines 10,10 Jahre alten Mädchens. **a**, **b** Schicht (Übersicht und Detailausschnitt) mit offener medianer und offener, nach anterior gewölbt verlaufender transversaler Gaumennaht sowie partiell offenen pterygopalatomaxillären Suturen, **c**, **d** Sowohl rechts- als auch linkslateral zeigt sich in der Sagittalebene eine weit geöffnete Sutura palatina transversa (*rote Pfeile*) mit geradem, senkrechten Verlauf von kranial nach kaudal, **e**, **f** Parallele Öffnung der Sutura palatina mediana und weit offene Sutura palatina transversa von kranial (**e**) und kaudal (**f**). Deutliche Vierteilung des knöchernen Gaumens

**Fig. 6:**
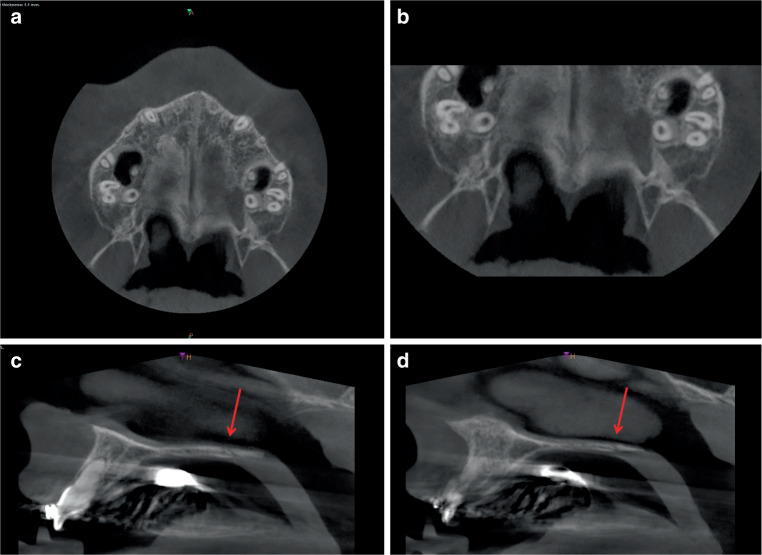
Horizontal (**a**, **b**) and sagittal slices (**c**, **d**) after combined rapid maxillary expansion/Delaire facemask (RME/DFM) treatment from a cone-beam computed tomography (CBCT) scan of a 12.8-year-old girl. **a**, **b** Transverse slice (overview and detail) with open median and open posteriorly curved transverse palatal suture and closed pterygopalatomaxillary sutures. **c**, **d** In the sagittal plane, the open transverse palatal suture (red arrows) runs obliquely/angulated on both sides from ventral–cranial to dorsal–caudal Horizontale (**a**, **b**) und sagittale Schichten (**c**, **d**) nach kombinierter RME/DFM(„rapid maxillary expansion/Delaire facemask“)-Behandlung aus der DVT (digitale Volumentomographie) eines 12,8 Jahre alten Mädchens. **a**, **b** Transversale Schicht (Übersicht und Detailausschnitt) mit offener medianer und offener, nach posterior gewölbt verlaufender transversaler Gaumennaht sowie geschlossenen pterygopalatomaxillären Suturen, **c**, **d** In der Sagittalebene verläuft die geöffnete Sutura palatina transversa (*rote Pfeile*) auf beiden Seiten schräg/anguliert von ventral-kranial nach dorsal-kaudal

**Fig. 7:**
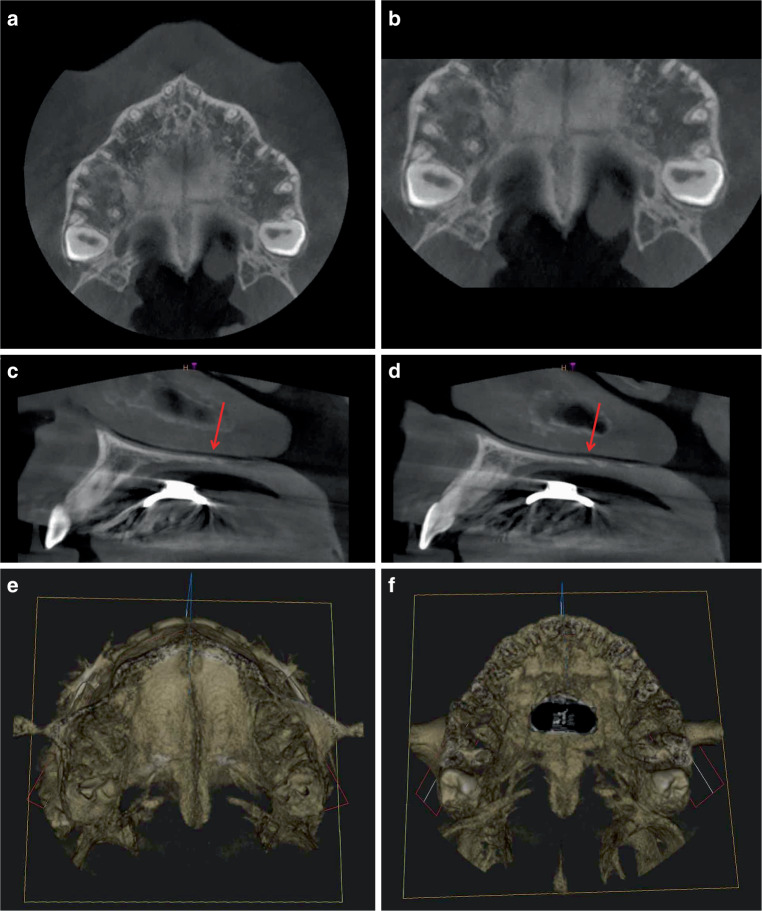
Horizontal (**a**, **b**) and sagittal slices (**c**, **d**) as well as 3D reconstructions with views from cranial and caudal (**e**, **f**) after combined rapid maxillary expansion/Delaire facemask (RME/DFM) treatment from a cone-beam computed tomography (CBCT) scan of a 14.7-year-old girl. **a**, **b** Layer (overview and detail section) with open median and transverse palatal suture. The transverse palatal suture runs straight with an offset, asymmetrical exit from the median palatal suture. The pterygopalatomaxillary sutures are closed. **c**, **d** In the sagittal plane, the opened transverse palatal suture (red arrows) runs obliquely/angulated on both sides from ventral–cranial to dorsal caudal. **e**, **f** Small opening of the median and transverse palatal sutures from cranial (**e**) and caudal (**f**). + Hyrax screw visible as an artefact in the caudal view Horizontale (**a**, **b**) und sagittale Schichten (**c**, **d**) sowie 3‑D-Rekonstruktionen mit Ansichten von kranial und kaudal (**e**, **f**) nach kombinierter RME/DFM(„rapid maxillary expansion/Delaire facemask“)-Behandlung aus der DVT (digitale Volumentomographie) eines 14,7 Jahre alten Mädchens. **a**, **b** Schicht (Übersicht und Detailausschnitt) mit offener medianer und transversaler Gaumennaht. Die Sutura palatina transversa verläuft gerade mit versetztem, asymmetrischem Abgang von der medianen Gaumensutur. Die pterygopalatomaxillären Suturen sind geschlossen, **c**, **d** In der Sagittalebene verläuft die geöffnete Sutura palatina transversa (*rote Pfeile*) auf beiden Seiten schräg/anguliert von ventral-kranial nach dorsal-kaudal, **e**, **f** Geringe Öffnung der Sutura palatina mediana und der Sutura palatina transversa von kranial (**e**) und kaudal (**f**). + Hyraxschraube als Artefakt in der Kaudalansicht

The comparison of the three patients reveals fundamental morphological differences in the transverse palatal sutures in both the transverse and sagittal planes: The CBCT of the 10.10-year-old patient shows an open, anteriorly curved transverse palatal suture in the transverse plane (Fig. 5a, b). On both sides, the sagittal plane shows a wide-open suture with a straight, vertical course from cranial to caudal (Fig. 5c, d). In the 12.8-year-old patient, the transverse palatal suture is curved posteriorly in the transverse plane (Fig. 6a, b) and runs obliquely from ventral–cranial to dorsal–caudal on both sides in the sagittal plane (Fig. 6c, d). In the oldest, 14.7-year-old patient, the transverse palatal suture is straight in the horizontal plane with an offset, asymmetrical departure from the median palatal suture (Fig. 7a, b), and in the sagittal plane, as in the second patient, it is oblique on both sides from ventral–cranial to dorsal–caudal (Fig. 7c, d).

Both pterygopalatomaxillary sutures are partially open in the lateral region only in the youngest patient with angulated alignment to the FM traction vector (Fig. 5a, b), and completely closed in the two older patients with perpendicular alignment to the FM traction vector (Figs. 6a, b, and 7a, b).

The incisive suture was never visible at all, which is tantamount to complete closure.

The 3D images of the 10.10-year-old patient (Fig. 5e, f) and the 14.7-year-old patient (Fig. 7e, f) show significant differences in the sutural opening widths in the cranial and caudal views: In the younger patient, the median and transverse palatal sutures are completely and wide open. On the contrary, the 14.7-year-old patient has much smaller openings of both palatal sutures.

### Cephalometric analysis

At treatment begin, patients in both groups showed maxillary retrognathia with mesiobasal jaw base relation. The findings did not differ at that point. After RME/DFM treatment, a significant improvement in the mesiobasal jaw relation due to sagittal–ventral displacement of the maxilla is only detectable in the first patient group (Table 2).

**Table 2:** Cephalometric measurements Kephalometrische Messungen

	SNA [°]			SNB [°]			ANB [°]		
	T1	T2	∆T2-T1	T1	T2	∆T2-T1	T1	T2	∆T2-T1
	M ± SD	M ± SD	M ± SD	M ± SD	M ± SD	M ± SD	M ± SD	M ± SD	M ± SD
	95% CI	95% CI	95% CI	95% CI	95% CI	95% CI	95% CI	95% CI	95% CI
	(LL, UL)	(LL, UL)	(LL, UL)	(LL, UL)	(LL, UL)	(LL, UL)	(LL, UL)	(LL, UL)	(LL, UL)
PG 1	78.17 ± 3.06	80.96 ± 3.67	2.78 ± 1.47	79.78 ± 3.28	80.62 ± 4.07	0.84 ± 1.85	−1.61 ± 2.42	0.32 ± 1.87	1.93 ± 1.88
Age < 12 years	76.65, 79.69	79.13, 82.78	2.05, 3.51	78.15, 81.41	78.60, 82.64	−0.08, 1.76	−2.82, −0.41	−0.61, 1.25	1.00, 2.87
*p* (intra)	–		*<* 0.001 ***	–		*0.071* ^*NS*^	–		*<* 0.001 ***
PG 2	79.85 ± 2.36	80.92 ± 2.61	1.08 ± 1.41	81.22 ± 2.61	81.51 ± 2.54	0.29 ± 1.65	−1.35 ± 1.92	−0.61 ± 1.45	0.75 ± 1.35
Age ≥ 12 years	78.42, 81.27	79.35, 82.50	0.23, 1.93	79.64, 82.79	79.97, 83.04	−0.70, 1.29	−2.51, −0.20	−1.48, 0.27	−0.07, 1.56
*p* (intra)	–		0.017 *	–		*0.535* ^*NS*^	–		*0.070* ^*NS*^
*p* (inter) < 12 vs ≥ 12	*0.010* ^*NS*^	*0.978* ^*NS*^	0.003 **	*0.203* ^*NS*^	*0.494* ^*NS*^	*0.402* ^*NS*^	*0.753* ^*NS*^	*0.146* ^*NS*^	*0.062* ^*NS*^

Skeletal–sagittal cephalometric measurements

*M* mean, *SD* standard deviation, *CI* confidence intervals and significance levels (* *p* < 0.05, ** *p* < 0.01, *** *p* < 0.001), *LL* lower limit, *UL* upper limit, *NS* not significant, *PG* patient group

### Cast analysis

#### Palatal width (transverse plane, Table 3)

The width increases are significant at all measurement levels except for the skeletal–basal level in PG2. In PG1, the increase is greater posteriorly than anteriorly at all levels (ratio of the mean values dental 0.91, gingival 0.69, and skeletal–basal 0.51). In contrast, PG2 showed a greater increase in width at all levels anteriorly than posteriorly (ratio of mean values dental 1.42, gingival 1.34, and skeletal–basal 2.84). A significant difference between the groups exists posteriorly at the skeletal–basal level (*p* = 0.024). The amount of width increase within the groups decreases between the levels from dental to skeletal–basal in an ascending manner in all comparisons.

**Table 3:** Dental cast analysis: palatal width (transverse plane), intragroup, and intergroup comparison Modellanalyse: Gaumenbreite (Transversalebene), Intragruppen- und Intergruppenvergleich

Measurement (mm)	T1	T2	∆T2-T1	*p* (intra)	*p* (inter)
	(M ± SD)	(M ± SD)	(M ± SD)		Group 1
	95% CI	95% CI	95% CI		vs
	(LL, UL)	(LL, UL)	(LL, UL)		Group 2
*All patients*					
54–64/14–24, dental	34.69 ± 2.63	37.91 ± 3.03	3.21 ± 1.98	< 0.001 ***	NA
	33.85, 35.53	36.94, 38.88	2.58, 3.85		
54–64/14–24, gingival	26.68 ± 2.41	29.28 ± 2.72	2.60 ± 1.86	< 0.001 ***	NA
	25.91, 27.45	28.41, 30.15	2.00, 3.19		
54–64/14–24, basal	13.09 ± 3.12	14.33 ± 3.25	1.24 ± 1.94	< 0.001 ***	NA
	12.10, 14.09	13.30, 15.37	0.62, 1.86		
16–26, dental	45.75 ± 3.44	48.63 ± 3.43	2.88 ± 2.67	< 0.001 ***	NA
	44.65, 46.85	47.53, 49.73	2.02, 3.73		
16–26, gingival	33.08 ± 2.70	35.75 ± 3.00	2.67 ± 2.22	< 0.001 ***	NA
	32.22, 33.94	34.79, 36.71	1.96, 3.38		
16–26, basal	22.70 ± 4.18	23.82 ± 4.41	1.12 ± 1.56	< 0.001 ***	NA
	21.36, 24.03	22.41, 25.23	0.62, 1.62		
*Group 1*					
54–64/14–24, dental	33.92 ± 2.77	36.94 ± 3.72	3.02 ± 1.80	< 0.001 ***	0.541 ^NS^
	32.63, 35.22	35.20, 38.69	2.18, 3.86		
54–64/14–24, gingival	26.35 ± 2.71	28.45 ± 3.32	2.10 ± 1.66	< 0.001 ***	0.093 ^NS^
	25.08, 27.62	26.89, 30.00	1.32, 2.88		
54–64/14–24, basal	12.37 ± 3.37	13.23 ± 3.33	0.86 ± 1.65	0.031 *	0.221 ^NS^
	10.79, 13.95	11.67, 14.79	0.09, 1.63		
16–26, dental	44.22 ± 3.46	47.58 ± 3.09	3.36 ± 2.56	< 0.001 ***	0.258 ^NS^
	42.60, 45.84	46.14, 49.03	2.16, 4.56		
16–26, gingival	31.73 ± 2.42	34.77 ± 2.78	3.04 ± 2.12	< 0.001 ***	0.306 ^NS^
	30.60, 32.87	33.47, 36.07	2.04, 4.03		
16–26, basal	20.34 ± 3.97	22.02 ± 4.34	1.68 ± 1.11	< 0.001 ***	0.024 *
	18.49, 22.20	19.99, 24.05	1.16, 2.19		
*Group 2*					
54–64/14–24, dental	35.46 ± 2.31	38.87 ± 1.76	3.41 ± 2.17	< 0.001 ***	0.541 ^NS^
	34.38, 36.54	38.05, 39.69	2.39, 4.42		
54–64/14–24, gingival	27.02 ± 2.07	30.11 ± 1.63	3.09 ± 1.95	< 0.001 ***	0.093 ^NS^
	26.05, 27.99	29.35, 30.87	2.18, 4.00		
54–64/14–24, basal	13.82 ± 2.74	15.44 ± 2.82	1.62 ± 2.17	0.003 **	0.221 ^NS^
	12.54, 15.10	14.12, 16.76	0.60, 2.64		
16–26, dental	47.28 ± 2.73	49.67 ± 3.51	2.40 ± 2.75	< 0.001 ***	0.258 ^NS^
	46.00, 48.55	48.03, 51.32	1.11, 3.68		
16–26, gingival	34.43 ± 2.30	36.74 ± 2.95	2.31 ± 2.31	< 0.001 ***	0.306 ^NS^
	33.35, 35.50	35.36, 38.12	1.23, 3.39		
16–26, basal	25.05 ± 2.91	25.61 ± 3.80	0.57 ± 1.77	0.167 ^NS^	0.024 *
	23.68, 26.41	23.84, 27.39	−0.26, 1.40		

Widths (in mm) in the anterior (54–64/14–24) and posterior (16–26) regions at three different levels of the maxilla. The dental width, the gingival width, and the basal width are shown

*M* mean, *SD* standard deviation, *CI* confidence intervals and significance levels (* *p* < 0.05, ** *p* < 0.01, *** *p* < 0.001), *LL* lower limit, *UL* upper limit, *NS* not significant, *NA* not applicable, *PG 1* and *PG 2* patient groups 1 and 2

#### Palatal length (sagittal plane, Table 4)

The increase in palatal length was significant in all three measurement sections in both patient groups, whereby the anterior, posterior, and total increase was greater in PG1 than in PG2. Relatively speaking, the posterior increases were greater than the anterior increases in both groups. There is a significant difference between the posterior (median *p* = 0.008, 5 mm right *p* = 0.007, 5 mm left *p* = 0.006) and total (median *p* = 0.003, 5 mm right *p* = 0.031, 5 mm left *p* = 0.002) groups, but not anterior.

**Table 4:** Dental cast analysis: palatal depth/length (sagittal plane), intragroup, and intergroup comparison Modellanalyse: palatinale Tiefe/Länge (sagittale Ebene), Intragruppen- und Intergruppenvergleich

Variable	T1	T2	∆T2–T1	*p* (intra)	*p* (inter)
	(M ± SD)	(M ± SD)	(M ± SD)		Group 1
	95% CI	95% CI	95% CI		vs.
	(LL, UL)	(LL, UL)	(LL, UL)		Group 2
*All patients*					
Anterior 3rd pair of palatal folds to incisor median	16.99 ± 1.80	18.09 ± 1.99	1.09 ± 0.95	< 0.001 ***	NA
	16.42, 17.57	17.45, 18.72	0.79, 1.40		
Anterior 3rd pair of palatal folds to incisor 5 mm right	17.02 ± 1.97	17.99 ± 2.08	0.97 ± 1.04	< 0.001 ***	NA
	16.39, 17.65	17.33, 18.66	0.64, 1.31		
Anterior 3rd pair of palatal folds to incisor 5 mm left	16.78 ± 1.93	17.83 ± 1.99	1.05 ± 0.98	< 0.001 ***	NA
	16.16, 17.40	17.20, 18.47	0.74, 1.37		
Posterior 3rd pair of palatal folds to tuber plane median	28.51 ± 3.20	30.50 ± 2.88	1.99 ± 1.31	< 0.001 ***	NA
	27.49, 29.54	29.58, 31.42	1.57, 2.40		
Posterior 3rd pair of palatal folds to tuber plane 5 mm right	28.48 ± 3.10	30.43 ± 2.86	1.95 ± 1.21	< 0.001 ***	NA
	27.49, 29.47	29.52, 31.35	1.56, 2.34		
Posterior 3rd pair of palatal folds to tuber plane 5 mm left	27.72 ± 5.42	29.75 ± 5.58	2.02 ± 1.36	< 0.001 ***	NA
	25.99, 29.46	27.96, 31.53	1.59, 2.46		
Total palatal lengths incisor to tuber plane median	45.51 ± 3.61	48.59 ± 3.33	3.08 ± 1.66	< 0.001 ***	NA
	44.35, 46.66	47.52, 49.65	2.55, 3.61		
Total palatal lengths incisor to tuber plane 5 mm right	45.50 ± 3.67	48.42 ± 3.48	2.92 ± 1.50	< 0.001 ***	NA
	44.32, 46.67	47.31, 49.54	2.45, 3.40		
Total palatal lengths incisor to tuber plane 5 mm left	44.50 ± 5.80	47.58 ± 6.00	3.08 ± 1.66	< 0.001 ***	NA
	42.64, 46.36	45.66, 49.50	2.55, 3.61		
*Group 1*					
Anterior 3rd pair of palatal folds to incisor median	16.64 ± 1.62	17.94 ± 1.99	1.30 ± 0.89	< 0.001 ***	0.171 ^NS^
	15.88, 17.40	17.01, 18.87	0.88, 1.71		
Anterior 3rd pair of palatal folds to incisor 5 mm right	16.57 ± 1.93	17.55 ± 2.10	0.97 ± 1.20	0.002 **	0.996 ^NS^
	15.67, 17.48	16.56, 18.53	0.41, 1.53		
Anterior 3rd pair of palatal folds to incisor 5 mm left	16.26 ± 1.70	17.53 ± 1.99	1.27 ± 0.97	< 0.001 ***	0.164 ^NS^
	15.46, 17.06	16.60, 18.46	0.82, 1.72		
Posterior 3rd pair of palatal folds to tuber plane median	26.30 ± 2.08	28.82 ± 2.11	2.52 ± 1.38	< 0.001 ***	0.008 **
	25.32, 27.27	27.83, 29.81	1.88, 3.17		
Posterior 3rd pair of palatal folds to tuber plane 5 mm right	26.31 ± 2.03	28.77 ± 2.08	2.46 ± 1.20	< 0.001 ***	0.007 **
	25.36, 27.26	27.79, 29.74	1.89, 3.02		
Posterior 3rd pair of palatal folds to tuber plane 5 mm left	26.35 ± 1.98	28.94 ± 2.10	2.59 ± 1.28	< 0.001 ***	0.006 **
	25.42, 27.27	27.96, 29.92	2.00, 3.19		
Total palatal lengths incisor to tuber plane median	42.93 ± 2.26	46.76 ± 2.97	3.82 ± 1.74	< 0.001 ***	0.003 **
	41.88, 43.99	45.37, 48.15	3.01, 4.64		
Total palatal lengths incisor to tuber plane 5 mm right	42.88 ± 2.53	46.31 ± 3.12	3.43 ± 1.57	< 0.001 ***	0.031 **
	41.70, 44.07	44.85, 47.77	2.69, 4.17		
Total palatal lengths incisor to tuber plane 5 mm left	42.61 ± 2.63	46.47 ± 3.06	3.86 ± 1.59	< 0.001 ***	0.002 **
	41.38, 43.84	45.04, 47.91	3.12, 4.61		
*Group 2*					
Anterior 3rd pair of palatal folds to incisor median	17.35 ± 1.93	18.24 ± 2.02	0.89 ± 0.98	< 0.001 ***	0.171 ^NS^
	15.64, 18.34	16.77, 19.54	0.43, 1.36		
Anterior 3rd pair of palatal folds to incisor 5 mm right	17.46 ± 1.96	18.44 ± 2.01	0.97 ± 0.89	< 0.001 ***	0.996 ^NS^
	15.66, 18.78	16.74, 19.77	0.62, 1.46		
Anterior 3rd pair of palatal folds to incisor 5 mm left	17.29 ± 2.05	18.13 ± 1.99	0.84 ± 0.97	0.001 **	0.164 ^NS^
	15.37, 18.70	16.40, 19.01	0.35, 1.32		
Posterior 3rd pair of palatal folds to tuber plane median	30.73 ± 2.50	32.18 ± 2.57	1.45 ± 0.99	< 0.001 ***	0.008 **
	28.96, 32.62	30.71, 33.82	0.92, 2.30		
Posterior 3rd pair of palatal folds to tuber plane 5 mm right	30.65 ± 2.39	32.10 ± 2.57	1.45 ± 1.02	< 0.001 ***	0.007 **
	28.95, 32.47	30.40, 33.75	0.95, 2.21		
Posterior 3rd pair of palatal folds to tuber plane 5 mm left	29.10 ± 7.24	30.56 ± 7.63	1.45 ± 1.22	< 0.001 ***	0.006 **
	28.89, 32.47	30.24, 34.27	0.82, 2.11		
Total palatal lengths incisor to tuber plane median	48.08 ± 2.78	50.42 ± 2.63	2.34 ± 1.20	< 0.001 ***	0.003 **
	45.97, 49.79	48.36, 52.49	1.97, 3.14		
Total palatal lengths incisor to tuber plane 5 mm right	48.12 ± 2.63	50.54 ± 2.41	2.42 ± 1.26	< 0.001 ***	0.031 **
	47.28, 49.63	48.45, 52.09	1.65, 3.37		
Total palatal lengths incisor to tuber plane 5 mm left	46.39 ± 7.39	48.68 ± 7.86	2.29 ± 1.35	< 0.001 ***	0.002 **
	46.69, 49.06	47.48, 52.49	1.42, 3.20		

Palatal depth/length (in mm) median and 5 mm right and left paramedian. The anterior length, the posterior length, and the total length are shown

*M* Mean, *SD* standard deviation, *CI* confidence intervals and significance levels (* *p* < 0.05, ** *p* < 0.01, *** *p* < 0.001), *LL* lower limit, *UL* upper limit, *NS* not significant, *NA* not applicable, *PG1* and *PG2* patient groups 1 and 2

## Discussion

CBCT (selected), lateral cephalograms and casts (complete) from patients with RME/DFM therapy were analyzed. Although only one CBCT was available in selected patients after treatment, it can be assumed that there was no relevant influence upon sutures immediately prior to therapy due to the shortness of the RME/DFM therapy. Therefore, opened sutures can be interpreted as a therapy effect.

### Structural and morphological age-related changes of the transverse sutures of the maxillary complex

The anatomical structures of the anterior palate are continuously discussed in the literature. Depending on the point of view, the existence of a premaxilla is assumed, which means that it can also acquire therapeutic relevance. While Lisson and Kjær [39] assume that the anterior region of the palate, which is formed by the incisive suture in adults, cannot be equated with the medial portion in the context of palatal development, this differentiation is no longer made in more recent publications, and the maxillary complex is subdivided into premaxilla, maxilla, and palatal segments [40]. This subdivision is based, among other things, on the presence of the oblique–transverse incisive suture, which is supposed to represent the demarcation of the premaxilla. This is no longer detectable at an older age and could not be identified in any CBCT of the present study.

According to Delaire [41], the premaxilla is one of the facial growth units, the growth of which depends, among other things, on the incisive suture. Although the ossification of the incisive suture begins between the 3rd and 4th year of life, it is still postulated that it is possible to change the premaxilla by applying force. An enlargement of the Os incisivum, which is underdeveloped in maxillary retrognathia, with the help of a protraction mask is only possible up to the age of seven [42].

Remmelink [43] performed a study on 9 macerated human skulls of subjects without cleft formations between 1–10 years of age. He, on the other hand, was unable to cause separation of the premaxilla from the maxilla from as early as 2 years of age, even when orthopedic forces were applied.

The incisive suture was not detectable in any of the examined CBCT scans. Since all patients in the cast analysis of this study were between 7 and 15.5 years old, it can be assumed that neither the orthopedic forces through the RME (transverse) nor through DFM (sagittal) had any effect on the region of a former incisive suture.

Not only the incisive suture, but also the median palatal suture and the transverse sutures of the maxillary complex—the transverse palatal and the paired pterygopalatomaxillary sutures—undergo structural and morphological age-related changes. Melsen and Melsen [44] and Melsen and Ousterhout [45] studied the postnatal development of the pterygopalatomaxillary region using human autopsy material. They described that disarticulation of the pterygopalatomaxillary suture is possible only at a very early age [45]. Already in the juvenile stage, the irregular bone surfaces of the suture between the palatine bone and the pterygoid processes of the sphenoid bone resulted in a complex morphology with strong interdigitation of the contact surfaces. Melsen and Melsen [44] suggested that except in early stages of postnatal development, the strong interdigitation exhibits marked resistance to vertical and horizontal displacement of the maxilla. In the present CBCT study, the paired pterygopalatomaxillary sutures are still partially open laterally only in the two youngest, 10.5- and 10.10-year-old patients. In these 2 patients, all palatal length increases are also greater than in the 4 older patients (Table 1). The inclination of the suture itself (angulated or perpendicular) appears to be irrelevant.

Also using human autopsy material, Melsen (60 individuals between 0–18 years) [46] and Persson and Thilander (24 individuals between 15 and 35 years) [47] studied the postnatal development of the palate. Melsen [46] described that the morphology changes during postnatal growth both in the transverse palatal and in the median palatal sutures. At birth, the suture was broad and slightly sinuous, but at the age of about 10 it developed into a typical squamous suture, in which the palatine part overlapped the maxillary part. In the lower part of the suture, which was the broadest, incipient digitation was seen to the age of 10. In a sagittal section, the oral portion of the transverse palatine suture forms an angle. This oblique, angulated course in the sagittal plane could be found in the 4 older patients (> 12 years) of the CBCT study. In the two youngest, 10-year-old patients, the transverse palatal suture was recognizably open and revealed that the cranial–caudal course was straight. After the age of 13–14, the suture became shorter and slightly wavy, and the connective tissue sheet between the two parts of the palate narrowed. After the ages of 15 in girls and 17 in boys the transverse and midpalatal sutures consisted of a narrow sheet of connective tissue with inactive osteoblasts.

Since the oldest patient in the present study is 15.5 years old and male, gender-specific differences do not play a role. Moreover, the gender distribution is even in the two groups. Persson and Thilander [47] described in their histological study that the transverse palatal suture begins to obliterate later than the posterior, but earlier than the anterior part of the median palatal suture and found no gender-specific differences.

In summary, it seems that age-dependent cascade-like obliteration occurs in the transverse sutures of the maxillary complex, which may have a significant influence on the therapy-related effects of the RME/FM appliance on palatal morphology.

### Differentiated consideration of RME and DFM effects on palate morphology

Since RME and DFM are applied consecutively, their therapeutic effects can be considered isolated from each other. The width changes described in the present study are congruent with the results of the RME study by Kinzinger et al. [35]: transverse widening of the maxilla after RME tends to be uniform in children but becomes V‑shaped (anterior > posterior) and less pronounced with increasing age and especially in adolescents from the age of 12. The morphology of the median palatal suture is not the only limiting factor in the opening mode. Age-related changes in the transverse palatal and pterygopalatomaxillary sutures are responsible for the quality of the transverse expansion of the median palatal suture and, thus, also for the morphological changes of the maxillary palate [35].

According to the results of the present study, wearing the face mask appears to cause a significant increase in palate length, particularly in the posterior region in the second phase of the combination treatment. Length increases can only occur at the pterygopalatomaxillary and the transverse palatal sutures.

The pterygopalatomaxillary sutures were still partially open in the 10-year-old patients. Those were older than described by Kinzinger et al. [35] in an earlier investigation where this only occurred in the youngest, 7.3-year-old patient. Since disarticulation of the pterygopalatomaxillary suture described by Melsen and Melsen [44] and Melsen and Ousterhout [45] and tested in cephalometric studies by Baccetti et al. [32] and Franchi et al. [33] is only possible in infancy, it can be assumed that its contribution to the different palatal length changes found in the dental cast analysis is small.

Therefore, treatment effects upon the transverse palatal sutures must cause most of the recorded palatal length changes. In the present RME/DFM-CBCT study, the transverse palatal sutures were completely open in all patients, whereas in the RME-CBCT study by Kinzinger et al. [35] they were only open until the age of 10.8. The two clinical examples also show that the opening width decreases significantly with increasing age. In his study of human skulls, Tschechne [48] described clear differences in the sutural growth of the transverse palatal suture until and from the age of 12. It can be assumed that with stagnant sutural growth from the age of 12, the obliteration tendency and, thus, the rigidity of the transverse palatal suture increases significantly. The sutural aging processes increase the resistance to the orthopedic forces applied by the facemask. This is probably the main reason why in the present study the length increases differ significantly between the two patient groups PG1 and PG2.

Since systematic reviews and meta-analyses [49–51] have shown that rapid maxillary expansion has no improving influence on the protraction effect of the DFM on the maxilla, the therapeutic effects in the present study can be clearly differentiated: the changes in width are essentially caused by initial RME treatment, while the changes in length are caused by the subsequent facemask treatment. The results of the length measurements on the dental cast and the sagittal measurements on the lateral cephalograms—significant ventral development of the maxilla only in PG1—are consistent and in this context confirm Delaire’s postulate that a forward tilt of the maxilla is only possible up to the age of 12 years [30, 31]. Furthermore, it confirms conclusions by Baccetti et al. [32] and Franchi et al. [33] from cephalometric studies about the effect of disarticulation of the palatal bone from the pterygoid process, which only occurs during early mixed dentition.

When interpreting the present measurement results, it should be borne in mind that these represent summation effects from natural growth and therapeutic effects. Since measurement data from comparable untreated patients were neither available from own comparison groups nor from historical growth studies in the necessary scope and the corresponding measurement distances, therapeutic effects were compared between the two treatment groups. To enable this, the total collective was divided into two groups with equal numbers and largely identical number of Hyrax screw activations. Due to the shortness of the treatment and study period, it may be assumed that growth effects can be neglected compared to therapy effects. In addition, the RME appliances for retention in the passive state and the duration of wearing the facemask were in situ for comparatively the same length of time (mean 6 months) in the two patient groups.

## Limitations of the study

A methodological limitation is that the cast analysis was carried out on digitized models and not on human skull preparations. Bony structures can only be recorded approximately in this way, as the mucosal thicknesses vary at different points in time.

## Conclusion

In the present study, the age-related effects on the morphology of the palate after a combined rapid maxillary expansion/Delaire facemask (RME/DFM) treatment were investigated for the first time. The most relevant changes were as follows:

### Lateral cephalograms

- A significant improvement of the mesiobasal jaw relation due to sagittal ventral displacement of the maxilla during treatment occurred only in younger patients (< 12 years) despite similar findings in both patient groups at the start of treatment.

### Cast analysis, transverse plane

- The palatal width increases after forced expansion are nearly always significant. While younger patients (< 12 years) showed a greater increase posterior than anterior, the opposite is found in older patients (> 12 years), where the expansion occurs clearly greater anterior than posterior and thus V‑shaped.

### Cast analysis, sagittal plane

- The palatal length increases are always significant. The increase is posterior and overall significantly greater in younger (< 12 years) than in older patients (> 12 years).

### CBCT data

- Age-dependency is strongly supported through cone-beam computed tomography (CBCT) datasets, which allow the conclusion that age-dependent morphological changes of the pterygopalatomaxillary and the transverse palatine sutures have a decisive influence on the effects of both RME and DFM therapy.
- The age-dependent sutural reactions represent a further main therapeutic effect of DFM treatment in addition to those described by Delaire and explain the different palate length changes before and after the age of 12.

If a maximum effect from RME/DFM treatment is desired, it should be started before the age of 12. Treatment success is age-dependent.

## Data Availability

All data supporting the findings of this study are available within the paper.
